# A Carboxyl-Modified Polyaniline Cathode for High-Performance Aqueous Zinc-Ion Batteries

**DOI:** 10.3390/molecules30234498

**Published:** 2025-11-21

**Authors:** Zhen Sun, Shijun Tang, Haixu Wang, Shiyu Liu, Xiang Cai

**Affiliations:** 1 Liaoning Key Laboratory of Development and Utilization for Natural Products Active Molecules, School of Chemistry and Life Science, Anshan Normal University, Anshan 114005, China; sunzhen@asnc.edu.cn (Z.S.); wanghx918@nenu.edu.cn (H.W.); 2 School of Light Industry and Chemical Engineering, Dalian Polytechnic University, Dalian 116034, China; tsj2216@163.com; 3 Hegang Science and Technology Intelligence Research Institute, Hegang 154100, China; liushiyu2005@163.com

**Keywords:** aqueous Zn-ion battery, cathode, polyaniline

## Abstract

Inherent conductivity and high redox activity endow polyaniline (PANI) with great potential to serve as a cathode material for aqueous zinc-ion batteries. However, compared with traditional strongly acidic electrolytes (pH < 1), its electrochemical performances are moderated in weakly acidic zinc salt electrolytes (pH > 3) because of spontaneous deprotonation. Herein, a carboxyl-modified PANI was designed and synthesized by introducing carboxyl groups at the para-position of the terminal benzene rings within the polymer chains. In this conjugated system, the electron density in the polymer chains was redistributed with a higher one around the substituent due to the electron-withdrawing effect of carboxyl groups and meanwhile carboxyl groups characterized by a proton donor render PANI achieve a proton-involved electrochemical reaction. Consequently, the carboxyl-modified PANI cathode, in a Zn//PANI cell, delivers an impressive specific capacity of 226 mAh g^−1^ along with excellent rata capability and cycling stability. This work presented some new insights into the molecule structure design of PANI-based polymers applied in advanced aqueous batteries.

## 1. Introduction

Aqueous zinc-ion batteries (AZIBs) represent one of the promising alternatives to Li-ion batteries, owing to the distinct advantages of zinc metal anode, including high theoretical capacity (820 mAh g^−1^), low redox potential (−0.76 V vs. the standard hydrogen electrode), and good compatibility with water [1,2,3,4]. As one of key components in rechargeable batteries, the design and optimization of cathode materials are crucial for achieving excellent electrochemical performance. Various inorganic materials (e.g., transition-metal oxide, sulfides, phosphate-based materials and Prussian blue analogs) have been explored as potential cathode candidates [5,6,7,8,9,10,11,12]. However, their practical application is hindered by slow Zn^2+^ diffusion kinetics and structural degradation caused by repeated Zn^2+^ insertion/extraction processes [13,14,15]. Therefore, it is crucial to investigate novel host cathode materials that are suitable for AZIBs.

Compared with the aforementioned electrode materials, conducting polymers possess higher charge carrier mobilities owing to their long π-electron conjugated structures, which would enable superior energy storage performance [16,17,18,19,20,21]. As one of the typical conducting polymers, polyaniline (PANI) has attracted significant attention because of its high theoretical capacity (294 mAh g^−1^) and multiple redox states [22,23,24,25,26,27]. However, in weakly acidic zinc salt electrolytes, the capacity of PANI gradually deteriorates during repeated redox cycles due to swelling and shrinking, along with associated deprotonation processes. The integration of two-dimensional materials can mitigate structural stress, but it cannot provide highly acidic environment for PANI, failing to prevent deprotonation. There are three typical oxidation states of PANI (Figure S1): the fully reduced state (leucoemeraldine base, LB), the half-oxidized state (emeraldine base, EB), and the fully oxidized state (pernigraniline base, PB) [28,29,30,31,32]. The electrical conductivity of PANI is achieved through doping by means of reversible protonation of EB, which induces the formation of a conductive polysemiquinone radical cationic (i.e., polaronic) state due to a reorganization of the electronic structure [29,31]. Thanks to its conjugated nature of chemical bonding and the long-range conjugated molecular structure, nitrogen atoms can generate radical cations at their N-sites during oxidation without changing the number of attached hydrogen atoms [30,32]. To maintain electrical neutrality, anions intercalate into the polymer chain similarly to the charge compensation process in supercapacitors, thereby improving cycling stability and rate performance [33,34]. Unfortunately, this mechanism seems to conflict with the irreversible deprotonation of PANI in weakly acidic electrolytes [35,36,37]. An effective strategy is to introduce acidic functional groups-such as Brønsted acid moieties including sulfonic, phosphonic, and carboxylic acid derivatives, covalently into the PANI backbone, self-doped PANI can be constructed. These acidic groups not only serve to balance the positive charges formed during the redox process via intrinsic doping but also act as proton reservoirs by storing H^+^ ions, thereby enhancing the proton concentration within the polymer on the electrode. This capability enables PANI to retain high electrochemical activity even in low-acid zinc salt electrolytes [38,39]. Nevertheless, these substituents induce a degree of structural distortion in the benzene rings due to steric effects, which compromises π-conjugation along the polymer backbone and destabilizes the radical cation state [31,40,41]. Moreover, structural distortion in conjugated system not only limits the exposure of active sites but also reduces the efficiency of electron transport along the main chain [42,43,44,45]. Recent studies on organic small molecules have demonstrated that materials featuring asymmetric charge distribution within extended π-conjugated structure exhibit enhanced redox activity and strengthened intermolecular interactions for improved stability [46,47]. However, the application of such structural features in PANI-based materials remains relatively limited.

Based on the above considerations, this study employed an electrochemical copolymerization approach to copolymerize aniline with p-aminobenzoic acid, aiming to selectively introduce the carboxyl groups at the para position of the benzene ring located at the polymer chains terminus, thereby enabling precise design and regulation of functionalized polymer structure. The electron-withdrawing effect of the substituent directs the charge distribution toward the carboxyl groups, thereby enhancing both the electronic conductivity and redox activity of the polymer, which ultimately leads to improved electrochemical performance. On the other hand, the carboxyl groups serve as a proton reservoir, supplying sufficient H^+^ ions to support continuous protonation of PANI and prevent its extensive deprotonation. As a result, C-PANI-p cathode delivers a high capacity of 226 mAh g^−1^ at 0.2 A g^−1^, an excellent rate capability with a capacity of 166 mAh g^−1^ at 10 A g^−1^, and a good cyclability of 88% over 3000 cycles with nearly 100% coulombic efficiency.

## 2. Results and Discussion

**Figure 1:**
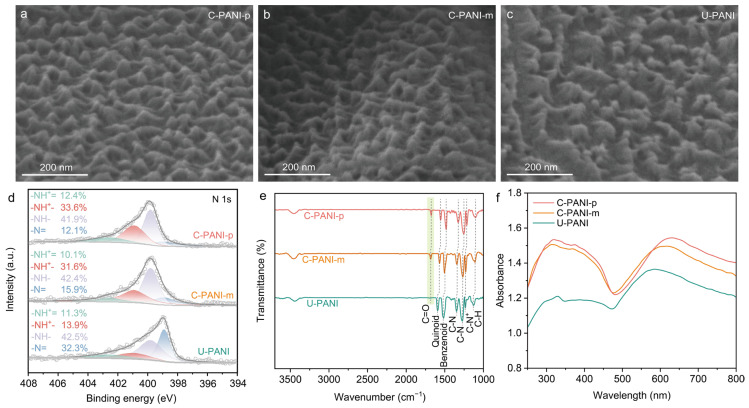
(**a**–**c**) SEM image of C-PANI-p, C-PANI-m, and U-PANI. (**d**) XPS N 1s, (**e**) FT-IR spectra, and (**f**) UV-Vis spectra of C-PANI-p, C-PANI-m, and U-PANI.

Two kinds of carboxyl-modified PANI cathodes were constructed through the electrochemical copolymerization of aniline with either p-aminobenzoic acid or m-aminobenzoic acid at a molar ratio of 4:1 on the EG substrates. The obtained electrodes were named C-PANIN-p and C-PANI-m, respectively. For comparison, the non-carboxyl-modified PANI (N-PANI) was also prepared using a similar electrochemical strategy in the absence of aminobenzoic acids. Figure 1a–c first present the morphology images of C-PANI-p, C-PANI-m, and N-PANI taken by a scanning electron microscope (SEM). A well-ordered array structure is observed for all electrodes. The chemical states of element N were further studied by X-ray photoelectron spectroscopy (XPS) technique. As shown in Figure 1d, four N-related components can be identified from the N 1s XPS spectra including the non-protonated imine (–N=) at 389.9 eV, the non-protonated amine (–NH–) at 399.8 eV, the protonated amine (–NH^+^–) at 399.8 eV, and the protonated imine (–NH^+^=) at 402.5 eV [20,32]. Compared to N-PANI, which exhibits a low N protonation level of 25.2% (the percentage of N^+^/N), the N protonation levels markedly increase to 46.0% and 41.7% for C-PANI-p and C-PANI-m, respectively, implying an improvement in electrical conductivity.

To confirm the contributions from the different components (ClO_4_^−^ and –COO^−^) to protonation level, the characteristic element (Cl, C, and N) contents were further quantificationally analyzed by ion chromatography (IC) and organic element analysis (OEA) methods. The detailed calculation procedures were presented in the Supporting Information. Table S1 shows the calculation results. Typically, in U-PANI, the protonated N is mainly from the doping effect of ClO_4_^−^. In contrast, the mass fraction of the C_6_H_5_NCO_2_ in C-PANIN-p was calculated to be 18.69%. As a result, together with the enhanced N protonation level as suggested by the XPS results, it can be concluded that the carboxyl groups in the polymer chains have a self-doping effect. The contribution ratios to the protonated N of C-PANIN-p are 58.36% and 41.64% for ClO_4_^−^ and –COO^−^, respectively. A similar result was also demonstrated in C-PANI-m, however with a higher self-doping proportion (55.53% for ClO_4_^−^ and 44.47% for –COO^−^). Fourier Transform Infrared spectroscopy (FT-IR) technique was used to investigate the evolution of the bonding environment in the three materials (Figure 1e). Both C-PANI-p and C-PANI-m display the absorption band associated with C=O stretching vibration, which is indicative of the presence of the carboxyl groups [24]. Compared to U-PANI, the characteristic absorption peak of PANI shifts toward lower wavenumbers, which is due to the additional protonation occurring and the increasing degree of charge delocalization on C-PANI-p and C-PANI-m backbone [27]. Notably, the peak shift is more pronounced in C-PANI-p. For C-PANI-m, the carboxyl groups can occupy two distinct positions along the polymer chain: the mid-chain and the chain terminus (Figure S2a,b). In the former case, the side-chain carboxyl groups attached to the benzene rings induce torsional strain, which can disrupt the planar conformation of the polymer backbones and decrease the extent of orbital overlap within the π-conjugated system. In the latter case, the carboxyl groups, acting as a bulky substituent, also favor chain termination during copolymerization, thereby positioning the carboxyl groups at the meta-position of the terminal benzene ring in the polymer chain [37,41]. In contrast, for C-PANI-p, the carboxyl groups are exclusively located in the para-position of the terminal benzene ring along the polymer chain (Figure S2c). The introduction of the carboxyl substituent onto the terminal benzene rings of the polymer chains facilitates the π-conjugated structure. According to resonance theory, the electron-withdrawing effect of the carboxyl groups induces a reduction in the electron density at the para position of the benzene ring, but not the meta-position, thereby facilitating electron migration along polymer chains towards the substituent. This electronic redistribution not only facilitates the coordination between the carboxyl groups and cations, but also enhances the stability of free radicals on the polymer chains. Moreover, the charge distribution within the π-conjugated backbone can reduce the energy gap between the lowest unoccupied molecular orbital (LUMO) and the highest occupied molecular orbital (HOMO), consequently enhancing electronic conductivity and electrochemical activity [48,49,50]. The red shift observed in the absorption band, as shown by UV-Vis spectroscopy (Figure 1f), confirms the reduction in the energy gap in C-PANI-p [41].

**Figure 2:**
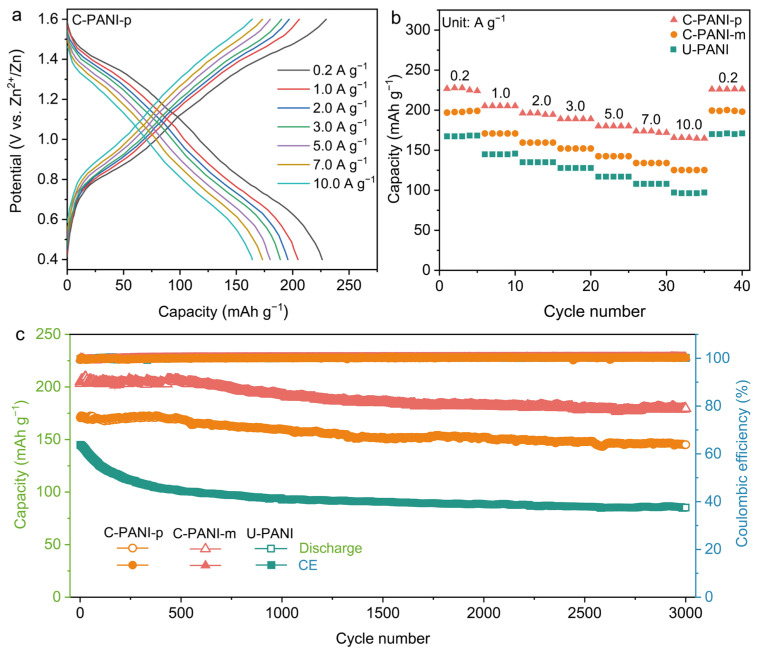
(**a**) GCD curves of C-PANI-p at various current densities. (**b**) Rate capability of C-PANI-p, C-PANI-m, and U-PANI at various current densities. (**c**) Long-term cycling stability of C-PANI-p, C-PANI-m, and U-PANI at 1 A g^−1^ for 3000 cycles.

The electrochemical performance of the synthesized PANI cathode materials was evaluated using a Zn anode and 9 M ZnCl_2_ aqueous electrolyte to validate the structural advantages for enhancing charge storage. Figure 2a and Figure S3 present the galvanostatic charge–discharge (GCD) profiles obtained at various current densities. C-PANI-p delivers a high capacity of 226 mAh g^−1^ at a current density of 0.2 A g^−1^, whereas the capacities achieved by C-PANI-m and U-PANI are only 197 and 167 mAh g^−1^, respectively. Moreover, even when the current density is increased to 10 A g^−1^, C-PANI-p maintains a capacity of 166 mAh g^−1^, retaining 73% of its initial capacity (Figure 2b), which is significantly higher than the 63% and 58% retention observed for C-PANI-m and U-PANI cathodes, respectively. The EG substrate shows negligible capacity (Figure S4), indicating that the electrochemical activity originates predominantly from the polymer active material. Long term cycling was evaluated at a current density of 1 A g^−1^ (Figure 2c). C-PANI-p exhibits a high capacity of 181 mAh g^−1^ and achieves a capacity retention of 88% (C-PANI-m: 83%, U-PANI: 58%) over 3000 cycles, with the coulombic efficiency (CE) remaining consistently close to 100%. Noticeably, the electrochemical performance of C-PANI-p clearly demonstrates the necessity of introducing a carboxyl group at the para position of the phenyl ring located at the terminus of the polymer chain. Furthermore, C-PANI-p exhibits superior performance compared to reported analogous PANI modified materials (Table S2) [21,22,35,38,39].

**Figure 3:**
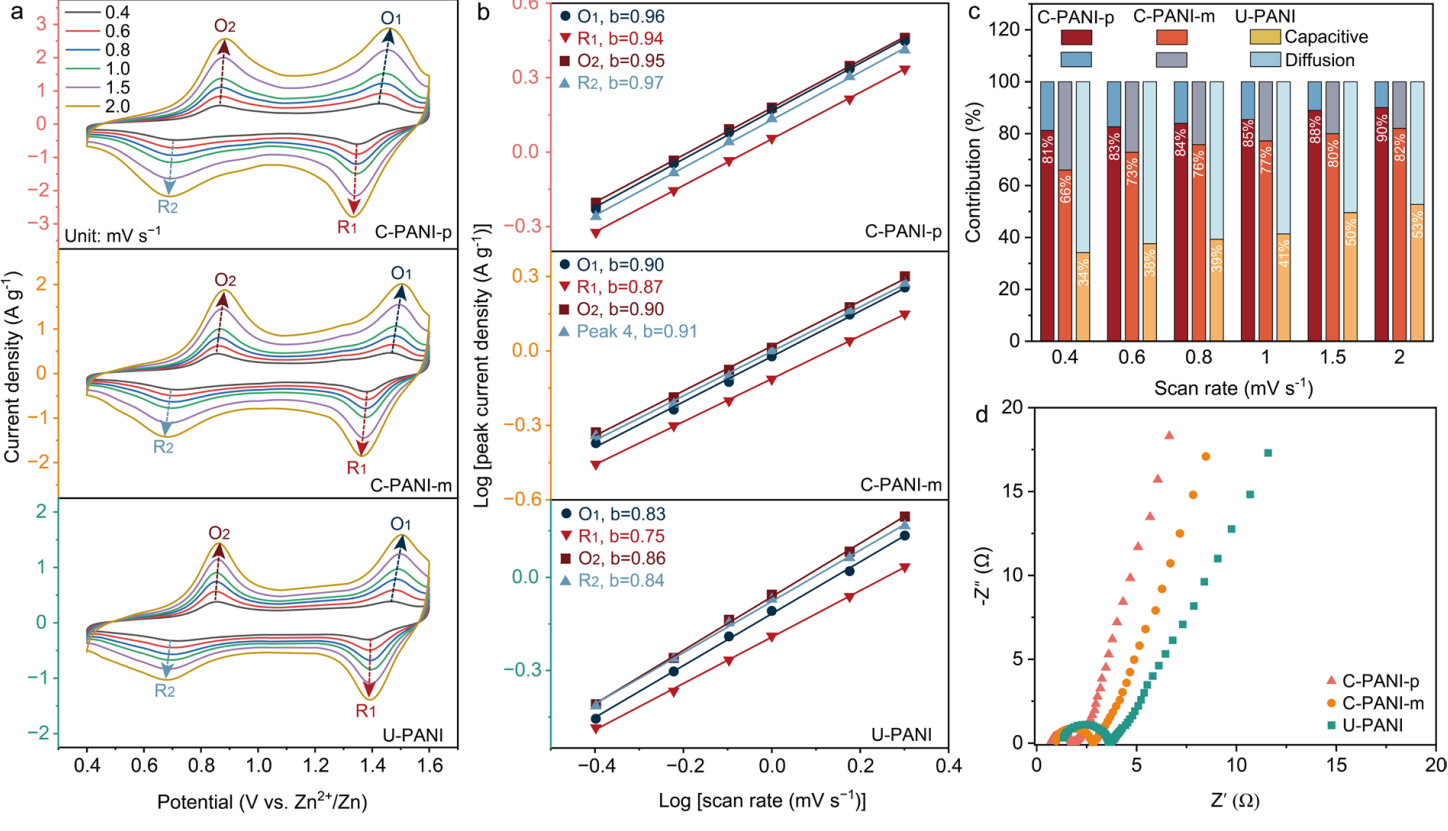
(**a**) CV curves electrodes at various scan rates. (**b**) The linear fits of lg(i) vs. lg(v) plots. (**c**) Capacitive and diffusion-controlled contributions at different scan rates. (**d**) Nyquist plots of PANI-PANI-p, C-PANI-m, and U-PANI.

The reaction kinetics of the prepared PANI electrodes were studied by cyclic voltammetry (CV) at scan rates ranging from 0.4 to 2 mV s^−1^, as presented in Figure 3a. The redox peaks observed at around 1.43/1.35 V (O_1_/R_1_) and 0.87/0.71 V (O_2_/R_2_) are characteristic of the redox reaction at quinone sites and the leucoemeraldine-emeraldine transition, respectively [39]. The peak current density (*i*) and the scan rate (*v*) of the CV profile should obey the relationship *i* = a*v*^b^, where a and b are variables. A b-value of 0.5 indicates diffusion-controlled behavior, whereas a b-value of 1.0 suggests capacitive-controlled behavior. The linear fitting analysis of the lg(i) vs. lg(v) curves yields calculated b-values for the prepared PANI materials (Figure 3b). Compared to C-PANI-m and U-PANI, C-PANI-p exhibits higher b-values of 0.96, 0.94, 0.95, and 0.97, indicating that its redox behavior is predominantly governed by capacitive contribution, which reflects faster reaction kinetics. Furthermore, the contribution percentage of surface-controlled process (*k*_1_*v*) and diffusion-controlled process (*k*_2_*v*^1/2^) at various scan rates were further calculated by the equation of *i* = *k*_1_*v* + *k*_2_*v*^1/2^. As shown in Figure 3c, these results indicate that the non-diffusion-controlled process predominates in C-PANI-p, contributing to its high capacity and excellent rate capability. Figure 3d shows that the Nyquist plot of C-PANI-p displays the steepest slope in the low-frequency region, indicating superior ion diffusion kinetics compared to C-PANI-m and U-PANI. Additionally, in the medium-to-high frequency range, C-PANI-p exhibits the smallest semicircular arc and the lowest intercept on the *x*-axis at high frequencies, corresponding to the lowest charge transfer resistance and ohmic resistance, respectively. These findings further confirm the superior rate capability of C-PANI-p.

**Figure 4:**
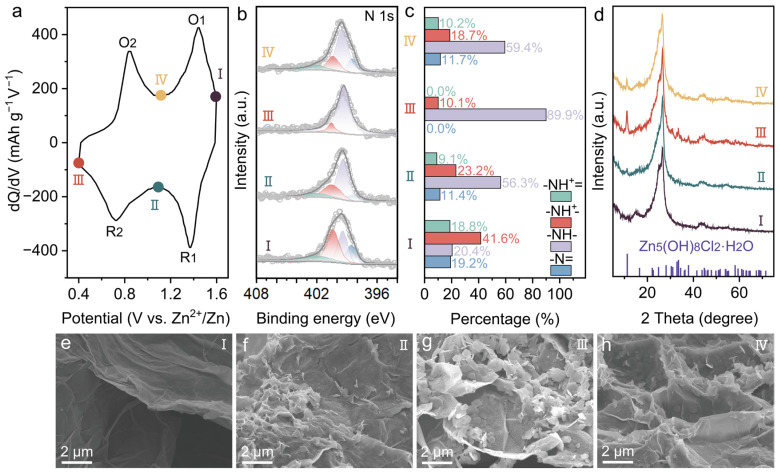
(**a**) The dQ/dV curve of C-PANI-p. (**b**) XPS N 1s of C-PANI-p at different states. (**c**) The calculated N contents of C-PANI-p at different states. (**d**) XRD patterns of C-PANI-p at different states. (**e**–**h**) SEM images of C-PANI-p at different states.

The energy storage mechanism of C-PANI-p was finally investigated. As shown in Figure 4a, the two redox pairs of the C-PANI-p electrode are analyzed at the characteristic points I-IV in the differential capacity (dQ/dV) curve. Ex situ XPS measurements were carried out to examine the compositional changes, and the corresponding results are displayed in Figure 4b,c. At the state of 1.6 V (stage I), the proportion of the oxidized N sites (i.e., -NH^+^=, -NH^+^-, and -N=) accounts for 79.6% of the total N content, closely resembling the pernigraniline state of PANI. Notably, at stage I, C-PANI-p exhibits an abundant presence of protonated nitrogen atoms (-NH^+^= and -NH^+^-), accounting for as much as 60.4%. This remarkable level of protonation endows C-PANI-p with excellent electrical conductivity in the pernigraniline state, thereby promoting a swift and efficient transformation between the pernigraniline and emeraldine states under high-potential conditions. Upon discharging to stage state II, the content of -NH^+^=, -NH^+^-, and -N= decreases, while that of -NH- increases. The proportion of the reduced N site (-NH-) reaches 56.3%, consistent with the emeraldine state of PANI. As the electrode was further discharged to state III, 89.9% of the nitrogen components is in the form of -NH-, indicating the effective reduction in -NH^+^=, -NH^+^-, and -N= species, corresponding to the transition from emeraldine to leucoemeraldine. Upon charging to state IV, the proportion of -NH- decreased to 59.4%, while the combined content of the other three nitrogen species (-NH^+^=, -NH^+^-, and -N=) increased to 40.6%. This observation demonstrates that a reversible conversion between oxidized nitrogen species and the reduced form occurs during the charging and discharging processes. Raman spectra (Figure S5) revealed that Zn(H_2_O)_2_Cl_4_^2−^, predominantly present in concentrated ZnCl_2_ solution, participates in the charge and discharge processes to counterbalance the positive charges on the polymer chains [26,51]. Once discharge begins, the protonated -NH^+^- and -NH^+^= are directly reduced to -NH- through accepting electrons, accompanied by the release of intermediate Zn(H_2_O)_2_Cl_4_^2−^ dopant. On the other hand, the reduction in unprotonated -N= requires its prior protonation through binding with H^+^ ions, after which it can be reduced to -NH-. Thus, the significance of -COO^−^ in enhancing the electrochemical performance of C-PANI-p is clearly demonstrated. The deprotonated -COO^−^ can readily interact with H^+^ ions present in the electrolyte. Consequently, the resulting -COOH functions as an internal proton reservoir, continuously supplying H^+^ to promote the protonation of -N=, thereby facilitating a rapid reduction process. During discharge, the potential gradually decreases, thereby attracting an increasing amount of H^+^ ions due to the accumulation of negative charge on C-PANI-p cathode surface. These factors collectively accelerate the protonation of -N=. Accordingly, the formation of Zn_5_(OH)_8_Cl_2_·H_2_O is associated with the insertion of H^+^ into C-PANI-p due to the presence of residual OH^−^ (Figure 4d). Furthermore, SEM images (Figure 4e–h) reveal the progressive accumulation and structural evolution of polygonal sheet-like hydroxide products during the discharge process. In the reverse charge process, the opposite reaction involves the oxidation of -NH-, whereby the reduced PANI returns to its original state. This is accompanied by the decomposition of Zn_5_(OH)_8_Cl_2_·H_2_O and the interaction of Zn(H_2_O)_2_Cl_4_^2−^ with protonated nitrogen atoms.

Based on the above discussion, as shown in Figure S6, the energy storage process of C-PANI-p can be primarily attributed to the redox reactions occurring between -NH- and -NH^+^-, coupled with the reversible insertion and extraction of Zn(H_2_O)_2_Cl_4_^2−^ from the electrolyte as well as the self-doping facilitated by the -COO^−^. These processes enable the reversible transitions between leucoemeraldine/emeraldine and emeraldine/pernigraniline states, thereby contributing to excellent electrochemical performance, including high capacity utilization, superior rate capability, and good cycling stability.

## 3. Materials and Methods

### 3.1. Materials

Graphite foil (GF) was obtained from SGL Carbon GmbH, Bonn, Germany. LiClO_4_ was purchased from Aladdin Chemicals (Shanghai, China). Other reagents were supplied by Sinopharm Chemical Reagent Co., Ltd. (Shanghai, China) and used as received, except for aniline which was distilled under reduced pressure prior to use.

### 3.2. Electrochemical Exfoliation of Graphite Foil (EG)

The electrochemical exfoliation was carried out in a two-electrode system, where a GF piece [0.8 cm (L) × 0.8 cm (W)] served as the working electrode and a Pt plate was used as the counter electrode. A constant potential of −10 V was applied for 25 s in an electrolyte solution containing 150 mg mL^−1^ LiClO_4_ dissolved in propylene carbonate (PC), enabling the production of EG under ambient conditions. Following exfoliation, the EG sample was sequentially rinsed with ethanol and deionized water, and subsequently immersed in 1 M HCl aqueous solution for 6 h. Afterward, the EG was thoroughly washed multiple times with deionized water and then subjected to freeze-drying.

### 3.3. Electrochemical Deposition of Polyaniline (PANI)

The carboxyl-modified polyaniline (C-PANI) electrodes were synthesized via a facile galvanostatic copolymerization of aniline and aminobenzoic acid on EG. The polymerization was conducted in an aqueous electrolyte solution containing 1 M HClO_4_, 0.04 M aniline, and either 0.01 M p-aminobenzoic acid or m-aminobenzoic acid. The standard calomel electrode (SCE) and GF were used as the reference and counter electrodes, respectively. Electrodeposition was performed at a current density of 0.5 mA cm^−2^ for 1 h (*Q* = 3.62 C), yielding electrodes labeled as C-PANI-p and C-PANI-m, respectively. After synthesis, the electrodes were thoroughly rinsed with deionized water and dried under vacuum at 60 °C overnight. The mass of electrodes was measured using a semi-microbalance (BT 25S, Sartorius, Göttingen, Germany) with a sensitivity of 0.01 mg, resulting in an active material loading of 0.8 mg cm^−2^. For comparison, non-carboxyl-modified polyaniline (U-PANI) was prepared using the same procedure, with 0.05 M aniline as the sole monomer in the 1 M HClO_4_ electrolyte.

### 3.4. Fabrication of the Carbon Film Coated Zn Anode

Super P carbon and polytetrafluoroethylene (PTFE) were mixed in a mass ratio of 4:1. Deionized water was added gradually, and the mixture was thoroughly stirred to achieve a homogeneous slurry. The slurry was then rolled into a thin film and uniformly coated onto a circular zinc substrate with a diameter of 12 mm. After coating, the carbon coated Zn anodes were vacuum-dried at 60 °C.

### 3.5. Materials Characterization

The morphologies of the samples were examined using a scanning electron microscope (SEM, SU 8010, HITACHI, Tokyo, Japan). X-ray photoelectron spectroscopy (XPS) analysis was performed on an XPS spectrometer (ESCALAB 250Xi, Thermo Scientific, Waltham, MA, USA) employing Al Kα radiation as the excitation source (λ = 8.34 Å). The Cl element content was measured using ion chromatography (IC, ICS-1100, Thermo Dionex, Bend, OR, USA). The C and N element contents were measured through organic elemental analysis (OEA, Vario EL cube, Elementar, Langenselbold, Germany). The XPS data were calibrated based on the C1s peak at 284.8 eV. Fourier transform infrared spectroscopy (FT-IR) was carried out using a Bruker VERTEX70 spectrometer (Mannheim, Germany). UV-Vis spectra were recorded using a Lambda 650S UV-Vis spectrometer (London, UK), with PANI deposited onto indium tin oxide (ITO) substrates to eliminate interference from EG-based absorption.

### 3.6. Electrochemical Tests

The electrochemical performance of C-PANI-p, C-PANI-m, and U-PANI [0.8 cm (L) × 0.8 cm (W) × 0.3 mm (H)] was investigated using Swagelok^®^-type cells (Figure S7) with titanium rods as current collectors and a carbon coated Zn plate as the anode, in conjunction with 9 M ZnCl_2_ aqueous electrolyte (100 μL). Electrochemical impedance spectroscopy (EIS) analysis of C-PANI-p, C-PANI-m, and U-PANI was conducted in a three-electrode system, employing a carbon coated Zn plate as the counter electrode and a saturated calomel electrode (SCE) as the reference electrode. Measurements were performed at the open-circuit potential over a frequency range of 0.01 Hz to 100 kHz with a perturbation of 10 mV. All electrochemical tests were carried out using a CHI 660E electrochemical workstation or a LAND-CT2001A battery testing system.

## 4. Conclusions

In summary, we have successfully introduced the carboxyl groups at the para-position of the terminal benzene rings within the polymer chains. The electron-withdrawing nature of the substituents effectively channels the charge distribution toward the carboxyl groups via a π-conjugated framework, thereby significantly rapidly boosting electron transport and redox reactivity, ultimately resulting in enhanced electrochemical performance. Furthermore, the carboxyl groups function as an intrinsic proton reservoir, maintaining a high proton concentration around the polymer matrix, which facilitates the efficient proton conduction process. These effects enable C-PANI-p cathode to deliver a high capacity (226 mAh g^−1^ at 0.2 A g^−1^), excellent rate capability (166 mAh g^−1^ at 10 A g^−1^), and a long cycle life (88% capacity retention after 3000 cycles at 1 A g^−1^). This work offers valuable insights into the rational design of PANI materials and provides significant guidance for achieving optimal high-performance outcomes in AZIBs.

## Data Availability

The data presented in this study are available on request from the corresponding author. The data are not publicly available due to commercial reasons.

## Supplementary Materials

The following supporting information can be downloaded at: https://www.mdpi.com/article/10.3390/molecules30234498/s1, Figure S1: Diagram showing the different protonation and oxidation degrees of PANI; Figure S2: Molecular structure of (a,b) C-PANI-m and (c) C-PANI-p in reduction state.; Figure S3: GCD curves of (a) C-PANI-m, and (b) U-PANI at various current densities; Figure S4: GCD curves of C-PANI-p and EG; Figure S5: Raman spectra of C-PANI-p at different states; Figure S6: The proposed redox mechanism of C-PANI-P in 9 M ZnCl_2_ electrolyte; Figure S7. Photograph and scheme of the electrochemical cell; Table S1: Elemental analysis data (wt%) of C-PANI-p, C-PANI-m, and CPANI-m; Table S2. Summary of self-doped PANI for AZIBs.
