# Supplementary material for: A Carboxyl-Modified Polyaniline Cathode for High-Performance Aqueous Zinc-Ion Batteries
Source: Molecules. 2025 Nov 21;30(23):4498. doi: 10.3390/molecules30234498 (PMC12693330; doi:10.3390/molecules30234498)
Supplement: Supplementary file 1 [file molecules-30-04498-s001.zip › molecules-3997005-supplementary.pdf]

A Carboxyl-Modified Polyaniline Cathode for High-Performance Aqueous Zinc-Ion Batteries

Zhen Sun [1], Shijun Tang [2], Haixu Wang [1], Shiyu Liu [3] and Xiang Cai [1,2,*]

[1] Liaoning Key Laboratory of Development and Utilization for Natural Products Active Molecules, School of Chemistry and Life Science, Anshan Normal University, Anshan 114005, China; sunzhen@asnc.edu.cn (Z.S.); wanghx918@nenu.edu.cn (H.W.)

[2] School of Light Industry and Chemical Engineering, Dalian Polytechnic University, Dalian 116034, China; tsj2216@163.com

[3] Hegang Science and Technology Intelligence Research Institute, Hegang 154100, China; liushiyu2005@163.com

* Correspondence: caixiang@dlpu.edu.cn

Supplementary Figures and Table

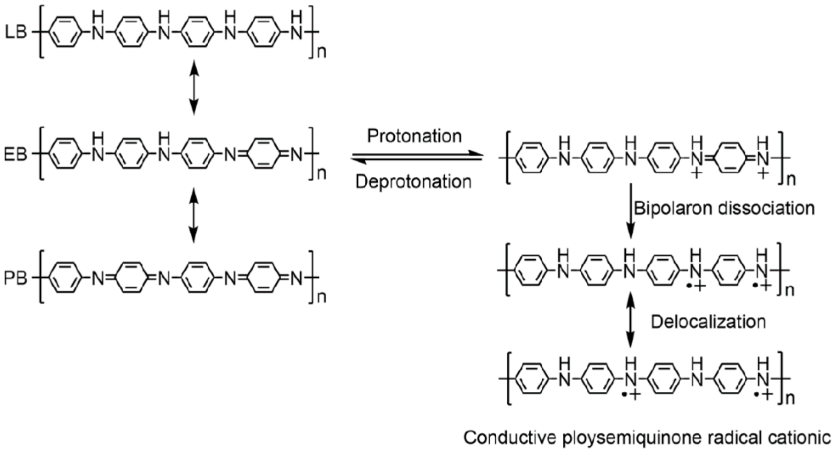

Figure S1. Diagram showing the different protonation and oxidation degrees of PANI.

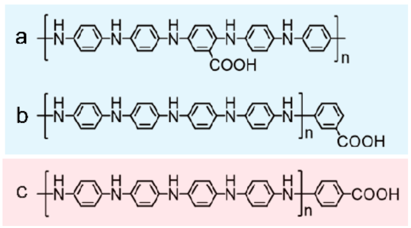

Figure S2. Molecular structure of (a and b) C-PANI-m and (c) C-PANI-p in reduction state.

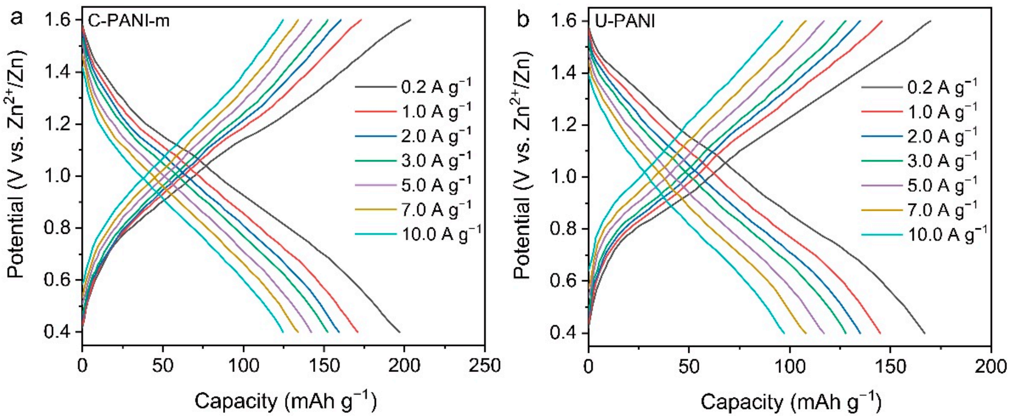

Figure S3. GCD curves of (a) C-PANI-m and (b) U-PANI at various current densities.

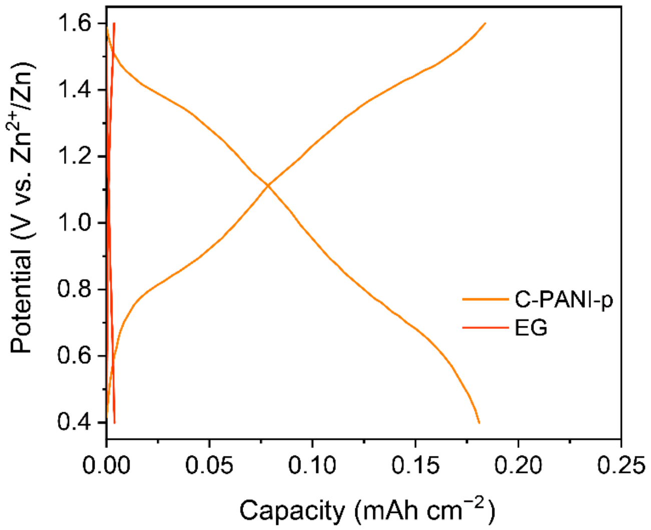

Figure S4. GCD curves of C-PANI-p and EG.

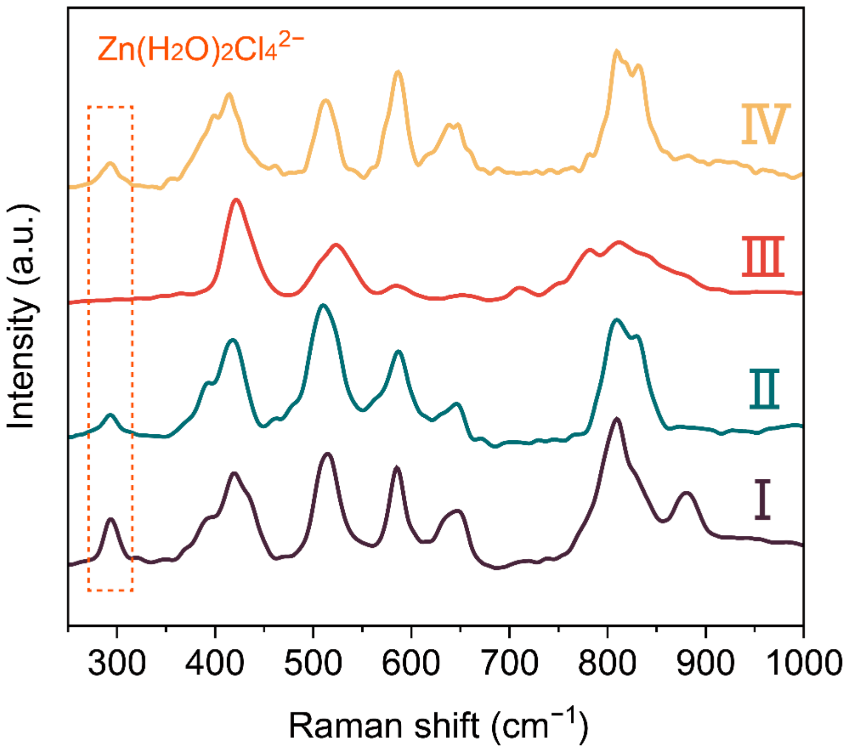

Figure S5. Raman spectra of C-PANI-p at different states.

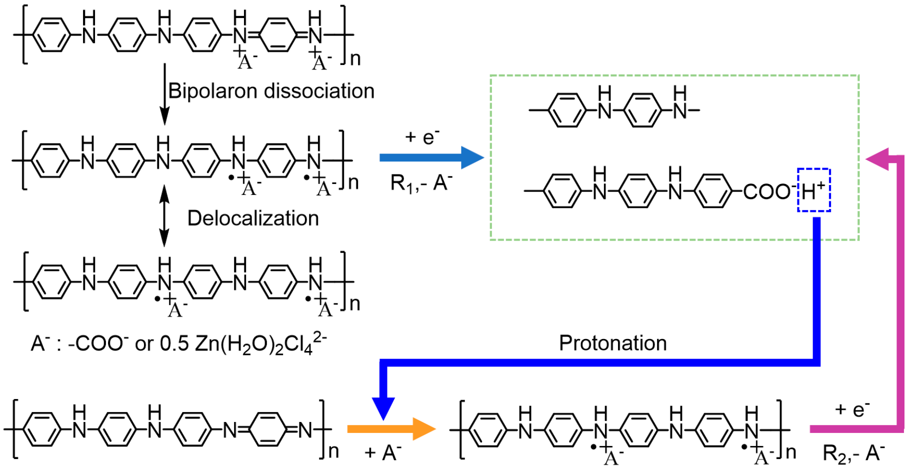

Figure S6. The proposed redox mechanism of C-PANI-P in 9 M $ZnCl_2$ electrolyte.

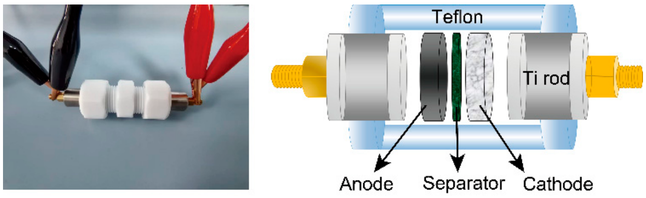

Figure S7. Photograph and scheme of the electrochemical cell.

Table S1. Elemental analysis data (wt%) of C-PANI-p, C-PANI-m, and CPANI-m.

Sample	Cl	C	N
C-PANI-p	4.88	56.33	10.63
C-PANI-m	4.27	55.58	10.49
N-PANI	5.38	44.74	8.71

Note

The protonation contributions of ClO_4^- and the aminobenzoic acid within the polymer chain were calculated based on the IC and the OEA results. Within the polymer chain, four distinct structural units are present, namely benzenoid C_6H_5N (91 g mol^{-1}), quinoid C_6H_4N (90 g mol^{-1}), and their doped forms $C_6H_5NClO_4$ (190.5 g mol^{-1}) and $C_6H_5NCO_2$ (135 g mol^{-1}), each present in varying relative proportions.

The weight percentages of $C_6H_5NClO_4$ in C-PANI-p is

$$\frac{4.88\%}{35.5}\times 190.5 = 26.19\%$$

The weight percentages of $C_6H_5NCO_2$ in C-PANI-p is

$$\left(\frac{56.33\%}{12}-\frac{10.63\%}{14}\times 6\right)\times 135 = 18.69\%$$

The weight percentages of $C_6H_5NClO_4$ in C-PANI-m is

$$\frac{4.27\%}{35.5}\times 190.5 = 22.91\%$$

The weight percentages of $C_6H_5NCO_2$ in C-PANI-m is

$$\left(\frac{55.58\%}{12}-\frac{10.49\%}{14}\times 6\right)\times 135 = 18.35\%$$

The weight percentages of $C_6H_5NClO_4$ in U-PANI is

$$\frac{5.38\%}{35.5}\times 190.5 = 28.87\%$$

The molar ratio of C and N in U-PANI is

$$\left(\frac{44.74\%}{12}\right):\left(\frac{8.71\%}{14}\right)\approx 6$$

Table S2. Summary of self-doped PANI for AZIBs.

Cathode	Electrolyte	Capacity ($mAh\ g^{-1}$, $A\ g^{-1}$)	Rate retention ($mAh\ g^{-1}$, $A\ g^{-1}$)	Cycling performance (cycle, $A\ g^{-1}$)
C-PANI-p This work	9 M $ZnCl_2$	226, 0.2	166, 10	88%, 3000, 1
PANI-S[S1]	1 M $ZnSO_4$	184, 0.2	130, 10	85%, 2000, 10
Tannin-PANI[S2]	3 M Zn(OTF)$_2$	116.5, 1.0	72, 10	97.7%, 350, 1.0
S-$Ti_3C_2T_x$/PANI[S3]	2 M $ZnSO_4$	262, 0.5	160, 15	64%, 5000, 15
t-CNTs-PA-PE[S4]	2 M $ZnSO_4$	238, 0.2	145, 10	78%, 1500, 10
SPANI[S5]	1.0 M PVA-Zn(CF_3SO_3)$_2$ gel	180.5, 0.5	136, 10	80%, 1000, 5

References

S1. Shi, H.Y.; Ye, Y.J.; Liu, K.; Song, Y.; Sun, X. A Long-Cycle-Life Self-Doped Polyaniline Cathode for Rechargeable Aqueous Zinc Batteries, *Angew. Chem. Int. Ed.*, **2018**, *57*, 16359-16363.

S2. Dong, H.; Wang, L.; Zhang, F.; Li, H.; Zhao, X.; Wei, W.; Kang, Y.; Yan, C.; Sang, Y.; Liu, H.; Wang, S. Green Proton Reservoirs of PANI for Disposable High-Performance Zinc-ion Batteries, *Nano Energy*, **2024**, *128*, 109768.

S3. Liu,Y.; Dai, Z.; Zhang, W.; Jiang, Y.; Peng, J.; Wu, D.; Chen, B.; Wei, W.; Chen, X.; Liu, Z.; Wang, Z.; Han, F.; Ding, D.; Wang, L.; Li, L.; Yang, Y.; Huang, Y. Sulfonic-Group-Grafted $Ti_3C_2T_x$ MXene: A Silver Bullet to Settle the Instability of Polyaniline toward High-Performance Zn-Ion Batteries, *ACS Nano*, **2021**, *15*, 9065-9075.

S4. Liu, Y.; Xie, L.; Zhang,W.; Dai, Z.; Wei, W.; Luo, S.; Chen, X.; Chen,W.; Rao, F.; Wang, L.; Huang, Y. Conjugated System of PEDOT:PSS-Induced Self-Doped PANI for Flexible Zinc-Ion Batteries with Enhanced Capacity and Cyclability, *ACS Appl. Mater. Interfaces*, **2019**, *11*, 30943-30952.

S5. Wang, Y.; Jiang, H.; Zheng, R.; Pan, J.; Niu, J.; Zou, X.; Jia, C. A Flexible, Electrochromic, Rechargeable Zn-Ion Battery Based on Actiniae-Like Self-Doped Polyaniline Cathode, *J. Mater. Chem. A*, **2020**, *8*, 12799-12809.
